# Lived experiences of nursing students regarding learning in large classes and its effects on teaching and learning at the University of Namibia

**DOI:** 10.4102/curationis.v45i1.2243

**Published:** 2022-01-10

**Authors:** Trymore B. Nhokwara, Daniel O. Ashipala, Medusalem H. Joel

**Affiliations:** 1 Department of Nursing Sciences, Faculty of Health Sciences, University of Namibia, Rundu, Namibia

**Keywords:** large classes, teaching, learning, undergraduate nursing students, lived experiences, effects

## Abstract

**Background:**

Whilst the number of students who choose to enrol at institutions of higher education continues to increase, class size remains a challenge. Therefore, institutions of higher education should continuously explore the challenges experienced by students regarding learning in large classes and devise strategies to address such challenges. The experiences of nursing students regarding learning in large classes and its effects on teaching and learning at the University of Namibia (UNAM) and in Namibia are not extensively researched.

**Objectives:**

The objective of this study was to explore and describe the experiences of nursing students regarding learning in large classes and its effects on teaching and learning at the UNAM.

**Methods:**

A qualitative explorative, descriptive and contextual research design was employed. Data collection was conducted using semi-structured interviews to ascertain the experiences of nursing students regarding learning in large classes and its effects on teaching and learning at the UNAM. Fifteen undergraduate nursing students from the Rundu campus participated in the study using a purposive sampling technique.

**Results:**

The analysis of data led to the emergence of the following themes namely: negative learning experiences, positive learning experiences and mechanisms for improvement. The findings had a negative effect on participants’ learning outcomes.

**Conclusion:**

Findings from this study indicated that participants were dissatisfied with the size of their classes as the learning environment was not conducive for teaching and learning.

## Introduction

Globally, large class size remains a problem which affects the effectiveness of teaching and learning in tertiary schools (Zimmerman 2019:9). A large class is considered to be over-populated when the number of students exceeds the optimum level such that it causes hindrance in teaching-learning process (Awoyemi 2016:5). On the other hand, Epri (2016:97) argued that there is no exact definition of a large class as it differs from country to country as well as from one teaching situation to another. However, an overcrowded large class is perceived to accommodate more than 50 students (Epri 2016:95).

Large class size is a major problem in the educational sector of developing nations, and therefore African nations have also been grappling with this problem, of which Namibia is no exception (Ayeni & Olowe 2016:65). Epri (2016:95) further stated that the overload of large class, in general, has a potential to negatively impact the academic performance of students. Admittedly, most tertiary institutions in New York City are faced with a challenge of large classrooms, which potentially affects the performance of students (Zimmerman 2019:15).

University of Namibia (UNAM) is the largest leading national institution of higher education in the country which was established by an Act of Parliament No. 18 of 31 August 1992 as recommended by the commission on higher education (Mwelwa et al. 2020:15). It is a diverse institution with a student population from 43 countries and from all continents. It has 12 campuses nationwide as well as 11 regional centres countrywide. The issue of large classroom and limited infrastructures has been a matter of concern for one of the UNAM’s satellite campuses located in the Kavango East Region (Rundu campus), where each class accommodates approximately 85–100 students. This study seeks to explore and describe the experiences of nursing students regarding learning in large classrooms and its effects on the teaching and learning at UNAM.

Primary beneficiaries of this study are the nursing students and lecturers. The study results can be used to solve large class size problems; hence students may acquire quality education through a good interaction with lecturers. Lecturers could benefit from this study by using alternative teaching methods when dealing with large classes. The study provided important information for the university authorities on how larger classes can be better taught to promote quality education.

## Problem statement

Generally, the phenomena of large class size in tertiary education are not only a negative situation for developing nations, but also for developed nations (Marais 2016:1; West & Meier 2020:2). The School of Nursing in response to increased public healthcare demands, increased student intakes each year which resulted in large classes. Currently, there is an alarming problem of large classes at UNAM, Rundu campus, School of Nursing. One class accommodates 85–100 students; it is difficult to imagine how effective teaching and learning takes place under one lecturer in such large class size (Barrett et al. 2019:72). Gomendio (2017:3) reported that lecturers may struggle to satisfy all learning needs of students with different interests, personalities and capabilities. Lecturers may also find it difficult to provide equal chances for the students to participate and practice and to give timely and effective feedback and evaluation (Zehra et al. 2015:1069). In addition, some of the students may find it difficult to catch up in overcrowded classrooms owing to noise making (Coleman, Dinkel & Huberty 2015:316). There is higher expectation from tertiary education to produce the needed critical social knowledge, attitudes and skills towards a just society (Ayeni & Olowe 2016:66). Failure to properly prepare student nurses during tertiary education as expected has the potential to affect the quality of healthcare services, which may lead to the gradual decline in the country’s quality of health (Asma & Hanane 2015:7).

## Aim

The aim of this article is to explore nursing students lived experience of being taught in a large class and its effect on teaching and learning at the UNAM.

## Research method and design

A qualitative method using an explorative and contextual study design was undertaken (Creswell 2014:19). The contextual design allowed the researcher to explore how people make sense of their surroundings and experiences to understand a phenomenon such as learning in large classes. An exploratory research design ascertained the in-depth lived experiences of participants who were exposed to learning in large classes.

## Research instrument

The study used a semi-structured interview which focused on exploring the lived experiences of nursing students in large classes and its effect on teaching and learning at the UNAM. The interview was facilitated through the use of open-ended questions which oriented the study as described by (Burns, Grove & Gray 2015:67).

## Research setting

This study took place at one public university satellite campus located in northern part of Namibia. The university is a premier institution of higher learning in Namibia that has 12 satellite campuses nationwide. This particular satellite campus is home to approximately 2319 students, of those, 1804 are full-time students and around 413 are distance students. It has three faculties, namely: Education, Economics and Management Sciences and Health Sciences, with an average class size of 85–120 students.

## Population and sample

In this study, the target population included all undergraduate nursing students at a satellite campus situated in north-east Namibia. According to the records obtained from the registrar office, there were a total of 405 full-time students registered in the School of Nursing. This constituted the entire population to be included in this study. A purposive sampling method, as highlighted by Brink, Van der Walt and Van Ransburg (2018:25) was used to select 15 undergraduate nursing students. Participants were selected based on the following inclusion criteria; undergraduate nursing students registered with Namibia Nursing Council (NNC) under Nursing Act no 8 of 2004 and students who were being taught in large classes in the second semester of 2020. Data were collected until saturation was achieved with the 15th participant.

## Data collection procedures

In this study, the data were collected by the researcher using semi-structured individual interviews with undergraduate nursing students who met the study criteria. The date, time and place of interview were confirmed with participants. The interviews were conducted between September 2020 and November 2020. Interviews were conducted in a quiet place and the participants were made comfortable for the duration of the study. The interview session lasted approximately 35–45 min. During the interview, the researcher took notes and used probing gestures to encourage a detailed exploration of the experience. Individual interviews were audiotaped with the permission from the participants.

## Data analysis

Data analysis continued concurrently with data collection because the researcher reflected on the raw data as these became available. All recorded interviews were then transcribed verbatim. The data analysis process followed the descriptions of thematic analysis (Maguire & Delahunt 2017:3352). During the first step, the researcher read through all the transcripts independently in order to obtain a general sense of the information, and in the second step, she highlighted all the participants’ statements that made a substantial point. In step three, all the substantive statements were listed, themes were clustered together, and arranged into codes. Each theme was allocated a provisional code. In the fourth step, the analyst went through all the transcripts and marked the statements with the codes allocated to the themes. As the process continues, the groupings of the themes were revised to include new ones and to combine those that related to one another as new insights emerged as the analysts were immersed with the data. At this stage, the two analysts compared their work and discussed the differences and similarities in order to reach consensus. In step five, the most descriptive wording for each theme was identified and themes were divided into main themes and sub-themes. Again, themes were considered and changed as necessary. In step six, the themes were compiled, whilst the seventh step entailed combining the data that belonged to each theme. The statements were marked with an interview number and the page number within the transcripts. At this stage, an independent coder was used to discuss the final themes.

## Ethical considerations

Ethical approval to conduct the study was obtained from the chairperson of the School of Nursing Research Committee of the Faculty of Health Sciences, University of Namibia (reference number SoNREC 15/2020). The researcher ensured that the participants’ right to privacy, the right to anonymity and confidentiality, the right to fair treatment and the right to protection from discomfort and harm were considered through the process (Burns et al. 2015:67). In addition, a written informed consent was obtained from anonymous participants prior to interviews. Participation in the study was entirely voluntary and participants had the right to withdraw at any given time without any penalties whatsoever. Additionally, data collected were only accessible to the researcher, independent coder and the two study supervisors. Participants were provided with a number which were used throughout the data collection process to ensure their anonymity and privacy. Participants were assured of their right to terminate participation in the study at any point without having to explain themselves or receive penalties for doing so.

## Trustworthiness

Trustworthiness of the entire study was assessed using the criteria proposed by Polit & Beck (2017:599), namely, credibility, transferability, dependability, and conformability. Credibility was achieved through prolonged engagement as well as through accurate and faithful description of a phenomenon (Polit and Beck 2017:599). The researcher spent three weeks in the field conducting interviews. Transferability was ensured through data saturation, purposive sampling, dense description of the study design and methods, and the findings were made available with supporting quotes from participants. Dependability was facilitated through literature control, prolonged engagement and member checking, and confirmability was ensured through triangulation of the data. The researcher practised the attitude of flexibility by writing down her own background and experience to avoid them from influencing the participants’ responses. This study used an independent coder to confirm the data collected through the interview guide, which was confirmed by the researchers to make sure that the data represented accurate information provided by the participants.

## Findings

### Description of participants

Participants were undergraduate students who were studying at the UNAM, Rundu Campus. All participants were under the age of 35 years. A sample consisted of seven males and eight female students of which five students each were from second, third and fourth year levels, respectively.

### Presentation of the study results

The three themes that emerged from the data analysis were: (1) negative learning experiences, (2) positive learning experiences and (3) mechanism for improvement. The description of themes and sub-themes in this study is given below.

#### Theme 1: Negative learning experiences

This theme reflects the students’ experiences of learning in a class with a large number of students. The five sub-themes which emerged from this theme are: (1) lack of participation by students; (2) poor concentration in large classrooms affects learning outcomes; (3) large classes allows for opportunities to cheat during tests and examinations; (4) large classes lead to poor lecture control and (5) large classes mean delayed assessment feedback.

**Sub-theme 1.1: Lack of voluntary student participation:** Students reported that not everyone contributed or participated in all class activities as each student relied on the other under given group assignments and presentations. One participant said:

‘Some students will not be able to contribute to the assignment or a presentation that is given because each one’s mind-set has just relied on another person.’ (P1, 28 years old, female)

Another participant added:

‘Some of them will even say if I am with serious students they are always punctual so that student will not participate, will only be relying on the serious ones.’ (P6, 32 years old, female)

**Sub-theme 1.2: Poor concentration in large classrooms affects learning outcomes:** Participants indicated that they had difficulty hearing and seeing the content taught, which contributed to poor concentration or focus and thus led to poor assessment results. One of the participants said:

‘So, for me not hearing the lecturer or not seeing what is being projected really affects learning.’ (P2, 25 years old, male)

Another participant said:

‘Even though the lecturer is teaching well, it will not produce good results as expected because those sitting behind will not be able to see what is on the projector and some of us understand well when we are reading on the projector.’ (P10, 28 years old, male)

A third participant added:

‘When people are making noise in the classroom, there are some points that one cannot get and the student will end up doing bad in the test or in examination.’ (P15, 32 years old, female)

**Sub-theme 1.3: Large classes provided opportunities to cheat during tests and examinations:** Participants highlighted that because of the large classroom sizes, there is insufficient space in the large class during tests or exams, allowing many students to copy from each other as they are sitting close together. Students revealed that the consequences of coping will be seen when the student graduates and goes into the field where they are expected to be competent. One of the participants’ said:

‘The classroom is very small … when we are sitting close to each other I have experienced that there is a possibility of copying.’ (P8, 35 years old, male)

Another participant said:

‘When you just pass because you were copying … it will end up affecting you because once you graduate … you are expected to be doing all the things correctly.’ (P2, 25 years old, male)

**Sub-theme 1.4: Large classes lead to poor lecture control:** Participants highlighted that it was very difficult for the lecturer to control the classroom so as to provide a suitable teaching environment during a lecture. One participant said:

‘Such a class cannot be controllable.’ (P4, 27 years old, female)

Another participant added:

‘It is very difficult for the lecturer to control a class with a large number of students and you find that the class will be very noisy throughout.’ (P11, 27 years old, female)

**Sub-theme 1.5: Large classes mean delayed assessment feedback:** In large classes, the marking of assessments takes longer because of the large numbers. This affects students, especially those who need a re-test. They do not get enough time to prepare for a retest or revision before proceeding to different chapter. This was highlighted by the students when they were asked to explain the effects of large classes on teaching and learning. One of the participants said:

‘It really takes time for the lecturer to finish marking and if it is a test it will become very difficult for the lecturer to finish marking and enter our marks.’ (P2, 25 years old, male)

Another participant commented:

‘So the tests don’t come on time and it is a problem because once the results comes back, there will be no more enough time for the lecturer to set a retest.’ (P3, 23 years old, male)

#### Theme 2: Positive learning experiences

This theme reflects students’ positive learning experiences in a class with a large number of students. One sub-theme that emerged from this theme is diversity of opinion is discussed next.

**Sub-theme 2.1: Diversity of opinion:** Students pointed out that being in a class with a large number of students had a positive impact because they were exposed to different opinions from different people which would be useful to their learning. One of the participants said:

‘Positive side I will say it’s quite a bit fine; you get to learn a lot from different people that have different opinions and mind set to one question.’ (P5, 24 years old, male)

A second student reported:

‘Someone can basically say something new and helpful which you as a student did not thick of …’ (P13, 23 years old, female)

#### Theme 3: Mechanism for improvement

This theme emerged from the participants’ responses when they were asked to express their opinions on what could be done to reduce the problem of large classes and its effects on teaching and learning at the UNAM. The four sub-themes which emerged from this theme are: (1) building more infrastructures; (2) employing more lecturers; (3) reducing the student intake and (4) speakers installed in teaching venues.

**Sub-theme 3.1: Building more infrastructures:** Some of the participants suggested that more classrooms should be built so that even though there are still many students they will be well accommodated. One participant said:

‘I think they should build more classes at the campus.’ (P5, 24 years old, male)

Another added:

‘They just have to expand the classrooms to a size that is quite bigger enough so that at least there is enough space inside that building.’ (P1, 28 years old, female)

In addition, a third participant said:

‘They should build more lecture theatres to accommodate those large numbers of students.’ (P10, 28 years old, male)

**Sub-theme 3.2: Employing more lecturers:** Students suggested that the university should provide more lecturers so that one class could be split into two groups and each group would have its own lecturer to prevent overcrowding and its effects. One participant reported:

‘I think they should employ more lecturers so that we can be taught in different groups, each group with each lecturer.’ (P4, 27 years old, female)

Another participant said:

‘The university should employ more lecturers to cater for those large numbers of students.’ (P10, 28 years old, male)

**Sub-theme 3.3: Redu cing the student intake:** Participants suggested a reduction in the total number of students per year to reduce the problem of large classes. One participant recommended:

‘They should reduce the number of students that they take per year to at least 40 to 50.’ (P9, 25 years old, female)

Another said:

‘In my own opinion, they should take fewer students per year.’ (P15, 32 years old, female)

A third participant suggested:

‘They should just recruit few students per year, at least not more than 50.’ (P7, 29 years old, male)

Participants suggested that the university should have two nursing intakes per year to reduce the problem of large classes. One participant suggested:

‘I think if they take students twice a year the classes will not be overloaded.’ (P2, 25 years old, male)

Another student added:

‘My suggestion is that they should recruit students twice a year.’ (P12, 25 years old, male)

**Sub-theme 3.4: Speakers installed at teaching venues:** Participants suggested the installation of speakers in the venues so as to improve the sound quality.

One participant said:

‘They should also put some speaker’s right round the classroom so that even if you are sitting at the back you will be able to hear nicely.’ (P3, 23 years old, male)

Another added:

‘I suggest that they should put speakers in the classrooms so that all the students can hear.’ (P14, 22 years old, male)

## Discussion of findings

The aim of this article is to explore nursing students’ lived experience of being taught in a large class and its effect on teaching and learning at the UNAM. This section presents the discussion of the findings with regard to their experiences under the following themes: negative learning experiences and positive learning experiences.

### Negative learning experiences

It emerged from the study that large class sizes have both negative and positive effects on learning. However, negative experiences outweighed the positive outcomes. Participants experienced a lack of participation by students, especially in group assignments and presentations because they relied on the next person to complete the task. Similarly, a study conducted by West & Meier (2020:3) assessing the impacts of overcrowded classrooms through the eyes of students ascertained that students tend not to participate in a large class because they would rely on others.

The participants in this study also highlighted that they had different ways of learning; some understood faster than others and had difficulties keeping up with the pace of the lecturer. Also, students were not successfully exposed to the content being taught because they could not see the projector or the board. This finding is in line with the study conducted by Aoumeur (2017:349) on the impacts of large classes in school, which found that slow learners tend to be ignored when the class is big and thus students will not get sufficient exposure to the content being taught.

Students also related that they could not concentrate in large classes as they could not see what was projected at the front of the classroom. They further indicated that they could not hear what the lecturer was saying because of class being very noisy and disruptive. A similar study conducted by Ay Ayeni and Olowe (2016:9) revealed that classes with a large number of students are very noisy and this affects students’ hearing and interrupts their concentration.

Some participants raised concerns that many students copied from each other in tests and examinations because of sitting so close together. Participants stated that the consequences of copying will be seen when someone is in the field and is expected to be competent. Also, they faced challenges with presentations; because there are so many of them and everyone has to present, this takes a lot of time that could be spent on other activities. These results are similar to those of a study conducted by Asma and Hanane (2015:17), who found that students tend to copy each other when the class is overcrowded and activities like presentations consume much of the time that students should be spending on other activities.

Students revealed that there was a delay in the completion of marking of their assessments which affected them in that they did not have time to revise their work and make corrections before going on to the next topic. In addition, they did not have enough time to prepare for the makeup test in case they needed it. This finding is in line with a study conducted by Dinama et al. (2016:71) on students’ academic performance in large classes, which revealed that lecturers face challenges with assessing students when class sizes are large.

It emerged from this study that in large classes, individuals will not receive attention from the lecturer on specific areas they find challenging, which affects their performance. Similarly, Nirashnee (2015:13) study described teachers’ experiences of teaching in overcrowded classrooms as stressful, stating that the lecturer cannot attend to each and every student’s problem when the class is big.

The study found that there are certain implications associated with large classes. The number of students can be overwhelming and it is very difficult for the lecturer to control such a class and provide a suitable teaching and learning environment. These findings are similar to those of a study conducted in Tanzania on the impact of overcrowded classrooms by Akech (2016:10), who reported that it is very difficult for the lecturer to control a class with a large number of students.

This study also revealed that there was no adequate space in the classroom to accommodate a large group of students which resulted in some students not having anywhere to sit, forcing them to walk around campus looking for chairs whilst missing out on some parts of the lesson. This was in line with the findings of a study performed by Zimmerman (2019:10) who stated that large class sizes have a negative effect on students’ performance. On the contrary, Dinama et al. (2016:80) highlighted a positive academic performance following parental involvement despite some densely populated classes.

### Positive learning experiences

On the other hand, some participants indicated having some positive experiences with large class sizes as it also emerged from the study of Adebogun (2015:59) on the strategies for coping with overcrowded classrooms. They mentioned that many students lead to many opinions and they obtain different views from different people on a certain topic of discussion, which is quite beneficial. Ndawo (2016) affirmed that this was achieved when lecturers make use of didactic teaching strategy as well as by creating interaction and thought-provoking activities which helps keep students awake to learn. On the contrary, Ayeni and Olowe (2016) discovered the lack of motivation and lack of participation as attributes of large classes.

## Strengths and limitations

The study provided broader understanding and insight on how students experienced learning in large classes and the effect that it had on their learning. In addition, the use of an explorative design enabled the participants to freely narrate and interpret their experiences and also make suggestions for improvements. Similarly, the use of purposive sampling allowed the researcher to select the sample based on participants’ knowledge of the phenomena being studied, which helped the researcher to obtain in-depth information. The results obtained in this study were collected from one of the satellite campuses of the UNAM. Consequently, it cannot be said that the findings of this study are representative of all satellite campuses at the UNAM. This limits generalisation. The sample size was relatively small, study was restricted to one setting only and the perceptions of lecturers were not explored in this study.

## Recommendations

Based on the findings of the study, the following recommendations are made:

- Further research to explore the experiences of lecturers who teach in large classes should be examined to understand fully the effects of large classes on teaching and learning.
- With the emerging online learning, lecturers at this campus should look into the possibility of using blended learning as the preferred method of teaching.
- Lecturers should receive training on innovative teaching methods to solve large class challenges.

## Conclusion

The aim of this study is to explore nursing students lived experience of being taught in a large class and its effect on teaching and learning at the UNAM. It is evident that participants in this study were dissatisfied with the size of their classes. Participants indicated that they experienced many challenges with learning in such large classes. The study results can be used to solve large class size problems; hence students could acquire quality education through a good interaction with lecturers. Findings from this study can be used to solve large class size problems; hence students could acquire quality education through a good interaction with their lecturers. In addition, lecturers could benefit from this study by using alternative methods such as microphone in order to address and mitigate poor sound associated with large classes. The findings of this study call for well-articulated plans and actions from campus management teams to address the challenges of large classes and their effects on teaching and learning.
